# Serum neutrophil gelatinase-associated lipocalin has an advantage over serum cystatin C and glomerular filtration rate in prediction of adverse cardiovascular outcome in patients with ST-segment elevation myocardial infarction

**DOI:** 10.1186/s12872-017-0514-5

**Published:** 2017-03-15

**Authors:** Olga L. Barbarash, Irina S. Bykova, Vasiliy V. Kashtalap, Mikhail V. Zykov, Oksana N. Hryachkova, Victoria V. Kalaeva, Kristina S. Shafranskaya, Victoria N. Karetnikova, Anton G. Kutikhin

**Affiliations:** 1 Research Institute for Complex Issues of Cardiovascular Diseases, Sosnovy Boulevard 6, 650002 Kemerovo, Russian Federation; 2 Kemerovo State Medical University, Voroshilova Street 22a, 650029 Kemerovo, Russian Federation

**Keywords:** ST-segment elevation myocardial infarction, Renal dysfunction, Glomerular filtration rate, Cystatin C, Neutrophil gelatinase-associated lipocalin

## Abstract

**Background:**

The aim of this study was to assess significance of serum neutrophil gelatinase-associated lipocalin (sNGAL) and cystatin C (sCC) in prediction of adverse cardiovascular outcome after ST-segment elevation myocardial infarction (STEMI).

**Methods:**

We recruited 357 consecutive patients who were admitted to the hospital within 24 h after onset of STEMI. On the 1st and 12th-14th day after hospital admission, we measured levels of sNGAL and sCC. We also determined presence of renal dysfunction (RD), defined as glomerular filtration rate < 60 mL/min/1.73 m^2^. After 3 years of follow-up, we performed a logistic regression and assessed the value of RD, sNGAL, and sCC in prediction of combined endpoint, defined as cardiovascular death or any cardiovascular complication.

**Results:**

RD, sCC level ≥ 1.9 mg/L, and sNGAL level ≥ 1.25 ng/mL on the 12th-14th day of hospitalization were associated with a 1.6-fold, 1.9-fold, and 2.9-fold higher risk of adverse cardiovascular outcome, respectively. Area under the ROC curve was the highest for the model based on sNGAL level compared to the models based on sCC level or RD presence.

**Conclusions:**

Measurement of sNGAL level in patients with STEMI on the 12th-14th day after hospital admission may improve prediction of adverse cardiovascular outcome.

## Background

According to the World Health Organization statistics, coronary artery disease (CAD) is a leading cause of death worldwide [1]. An estimated 7.4 million people died from CAD in 2012, representing 11.2% of all global deaths [1]. In the Russian Federation alone, there were 597,921 deaths from CAD, which is the highest number amongst all countries included into analysis [1].

A number of investigations revealed a significant association of renal dysfunction [RD, defined as glomerular filtration rate (GFR) < 60 mL/min/1.73 m^2^] with a high risk of cardiovascular death or acute cardiovascular events [2–4]. Moreover, RD is significantly associated with an adverse cardiovascular outcome in patients with CAD [5]. A critical decrease in GFR and albuminuria commonly occur at the late stage of chronic kidney disease (CKD) when > 30% of nephrons are affected [6]. However, there is a crucial need in novel, highly sensitive and specific markers of RD at the early stages of CKD. Recently, serum cystatin C (sCC) and serum neutrophil gelatinase-associated lipocalin (sNGAL) were suggested as the promising candidates [7, 8].

It is known that CC arises in all nucleated cells and is one of the most important endogenous inhibitors of cysteine proteinases whilst NGAL is produced by tubular epithelial cells and neutrophils in response to inflammation or ischemia, inhibiting bacterial growth and inducing epithelial cell proliferation [9]. The diagnostic value of sCC and sNGAL was shown for acute kidney injury, progression of CKD, and acute cardiorenal syndrome [10, 11]. Moreover, there is growing evidence of sCC and sNGAL importance in atherosclerosis and myocardial remodeling [12, 13]. In addition, sCC and sNGAL are associated with the risk factors of atherosclerosis [14, 15].

We carried out this study with the aim to investigate the value of sCC and sNGAL in prediction of an adverse cardiovascular outcome after ST-segment elevation myocardial infarction (STEMI).

## Methods

We recruited 357 patients who were admitted within 24 h of STEMI onset to Research Institute for Complex Issues of Cardiovascular Diseases (Kemerovo, Russian Federation) in 2012–2013. The study was performed in accordance with the principles of Good Clinical Practice and the Declaration of Helsinki. The local ethical committee approved the study and all the participants provided written informed consent after a full explanation of the study was given to them.

The criteria of inclusion into the study were 1) age > 18 years; 2) diagnosis of STEMI according to the European Society of Cardiology (ESC) Guidelines [16]; 3) written informed consent to participate in the study. Criteria of exclusion were 1) age < 18 years; 2) past medical history of cancer, concomitant autoimmune and/or mental disorders; 3) recurrent MI after percutaneous coronary intervention (PCI) or coronary artery bypass graft (CABG) surgery.

Stable angina, congestive heart failure, arterial hypertension, hypercholesterolemia, and diabetes mellitus were diagnosed according to ESC guidelines on the management of stable CAD [17], ESC guidelines for the diagnosis and treatment of acute and chronic heart failure [18], ESH/ESC Guidelines for the management of arterial hypertension [19], ESC/EAS Guidelines for the management of dyslipidemias [20], and ESC/EASD Guidelines on diabetes, pre-diabetes, and cardiovascular diseases [21], respectively. Smoking, body mass index, past medical history of MI or stroke, and family history of CAD were defined using the medical records. Clinicopathological features of the patients are represented in Table 1.

**Table 1:** Clinicopathological features of the patients, *n* = 357

Feature	Value
Female gender, n (%)	99 (27.7)
Mean age, years (95% confidence interval)	61.3 (59.9–62.6)
Stable angina, n (%)	176 (49.3)
Congestive heart failure, n (%)	75 (21.0)
Arterial hypertension, n (%)	301 (84.3)
Hypercholesterolemia, n (%)	87 (24.4)
Diabetes mellitus, n (%)	60 (16.8)
Smoking, n (%)	180 (50.4)
Body mass index > 25 kg/m^2^, n (%)	265 (74.2)
Past medical history of myocardial infarction, n (%)	65 (18.2)
Past medical history of stroke, n (%)	31 (8.7)
Family history of coronary artery disease, n (%)	91 (25.5)

Selective coronary angiography was performed within the first hours after hospital admission using GE Healthcare Innova 3100 Cardiac Angiography System (General Electric Healthcare). Colour duplex screening of the extracranial arteries (ECA) and lower extremity arteries (LEA) was performed on the 5th-7th day after hospital admission in all patients using the cardiovascular ultrasound system Vivid 7 Dimension (General Electric Healthcare) with a 5.7 MHz linear array transducer (for ECA), a 2.5–3 MHz curved array transducer, and a 5 MHz linear array transducer (for LEA). An extent of arterial stenosis was assessed in B regimen and by dopplerography (visualizing the local haemodynamics in the stenosis zone). Common and internal carotid arteries, vertebral, and subclavian arteries were visualized from both sides during the ECA screening; common and deep femoral arteries, popliteal, anterior and posterior tibial arteries were visualized from both sides during the LEA screening. The intima-media thickness (IMT) of common carotid artery was measured in the automatic mode (the value < 1 mm was considered normal). Polyvascular disease was defined as an increase in IMT ≥ 1 mm or ECA and/or LEA stenosis.

The preferable methods of myocardial reperfusion were defined in the shortest terms and included PCI or systemic thrombolytic therapy (TLT). Myocardial revascularization was not conducted when technical problems occurred or in patients with complex coronary anatomy or those with contraindications to TLT or PCI. All patients received the standard therapy of unfractionated heparin, aspirin, clopidogrel, angiotensin-converting enzyme inhibitors, beta-blockers, and statins. Long-acting nitrates, calcium channel blockers, diuretics, inotropic and antiarrhythmic drugs were prescribed if needed.

Serum creatinine level was measured at hospital admission and before hospital discharge with the further calculation of GFR by Modification of Diet in Renal Disease (MDRD) formula. In the case of in-hospital death, the final level of serum creatinine was taken into account. RD was defined as GFR < 60 mL/min/1.73 m^2^. The levels of sCC and sNGAL were measured on the 1st and 12th-14th day after hospital admission by enzyme-linked immunosorbent assay using the respective kits of R&D Systems according to the manufacturer’s protocols. Reference values for sCC were 0.52–0.90 mg/L and 0.56–0.98 mg/L for females and males, respectively. Reference values for sNGAL were 0.037–0.106 ng/mL.

In-hospital case fatality rate was 10.4% (37/357 patients). The prevalence of in-hospital non-lethal cardiovascular complications is represented in Table 2. After 3 years of follow-up, we collected data from 87.8% (281/320) discharged patients. Follow-up was conducted by a telephone-based interview. Cardiovascular death, recurrent MI, stroke, hospital admission due to unstable angina, and acute decompensated heart failure were considered as an adverse cardiovascular outcome, or the study endpoints. The prevalence of the study endpoints is represented in Table 3.

**Table 2:** In-hospital non-lethal cardiovascular complications, *n* = 357

Complication	Value
Early postinfarction angina, n (%)	50 (14.0)
Recurrent myocardial infarction, n (%)	18 (5.0)
Stroke, n (%)	2 (0.6)
Arrhythmia or heart block, n (%)	96 (26.9)
Any non-lethal cardiovascular complications, n (%)	166 (46.5)

**Table 3:** Study endpoints after 3 years of follow-up, *n* = 281

Study endpoint	Value
Cardiovascular death, n (%)	43 (15.3)
Recurrent myocardial infarction, n (%)	40 (14.2)
Stroke, n (%)	12 (4.3)
Hospital admission due to unstable angina, n (%)	81 (28.8)
Acute decompensated heart failure, n (%)	23 (8.2)
Combined endpoint, n (%)	199 (70.8)

Statistical analysis was performed using MedCalc (MedCalc Software) and SPSS (IBM). A sampling distribution was assessed by D’Agostino-Pearson test. Descriptive data were represented by median, interquartile range (25th and 75th percentiles), mean, and standard deviation of the mean. Two independent groups were compared by Mann-Whitney *U*-test. An adjustment for multiple comparisons was performed using false discovery rate (FDR). *P*-values, or *q*-values if FDR was applied (*q*-values are the name given to the adjusted *p*-values found using an optimized FDR approach), ≤ 0.05 were regarded as statistically significant. For multivariate analysis, we performed a stepwise linear logistic regression using forward Wald method with the plotting of the receiver operating characteristic (ROC) curve and further calculation of the area under the curve (AUC). Cut-off levels for sCC and sNGAL were defined according to the linear logistic regression to determine the optimal predictive values but were not linked to GFR.

## Results

At hospital admission, all the patients were divided into two groups, with (*n* = 104, 29.1%) and without (*n* = 253, 70.9%) RD. The same stratification was carried out before hospital discharge [*n* = 86 (24.1%) and *n* = 271 (75.9%) patients with and without RD, respectively].

Medians of sCC and sNGAL levels on the 1st day after hospital admission were 1.21 (0.89–1.63) mg/L and 1.33 (0.36–1.91) ng/mL, respectively. Medians of sCC and sNGAL levels on the 12th-14th day after hospital admission were 1.50 (1.02–1.90) mg/L and 1.63 (1.25–2.62) ng/mL, respectively.

Patients with RD at hospital admission had significantly higher levels of sCC on the 1st and 12th-14th day after hospital admission [1.76 (1.06–1.96) and 1.75 (1.15–2.16) mg/L, respectively) compared to those without RD [1.16 (0.86–1.34) and 1.31 (0.95–1.66) mg/L], *p* = 0.037 and 0.001, respectively (Table 4). Patients with RD at hospital discharge had significantly higher levels of sCC [1.74 (1.29–2.17) mg/L] and sNGAL [1.93 (1.55–2.52) ng/mL] on the 12th-14th day after hospital admission in comparison with those without RD [1.41 (0.96–1.7) mg/L and 1.53 (1.18–2.62) ng/mL], *p* = 0.024 and 0.031, respectively (Table 5). Regarding all other comparisons, we did not find any significant differences.

**Table 4:** Concentrations of serum cystatin C and neutrophil gelatinase-associated lipocalin in patients with and without renal dysfunction at hospital admission, *n* = 357

Feature	Renal dysfunction at hospital admission, *n* = 104	No renal dysfunction at hospital admission, *n* = 253	*P* value
Serum cystatin C on the 1st day after hospital admission, mg/L	1.76 (1.06–1.96)	1.16 (0.86–1.34)	0.037
Serum cystatin C on the 12th-14th day after hospital admission, mg/L	1.75 (1.15–2.16)	1.31 (0.95–1.66)	0.001
Serum neutrophil gelatinase-associated lipocalin on the 1st day after hospital admission, ng/mL	1.41 (1.01–1.82)	1.36 (1.08–1.64)	0.95
Serum neutrophil gelatinase-associated lipocalin on the 12th-14th day after hospital admission, ng/mL	1.78 (1.43–2.12)	1.85 (1.65–2.06)	0.68

**Table 5:** Concentrations of serum cystatin C and neutrophil gelatinase-associated lipocalin in patients with and without renal dysfunction before hospital discharge, *n* = 357

Feature	Renal dysfunction before hospital discharge, *n* = 86	No renal dysfunction before hospital discharge, *n* = 271	*P* value
Serum cystatin C on the 1st day after hospital admission, mg/L	1.27 (1.04–1.69)	1.12 (0.95–1.4)	0.14
Serum cystatin C on the 12th-14th day after hospital admission, mg/L	1.74 (1.29–2.17)	1.41 (0.96–1.7)	0.024
Serum neutrophil gelatinase-associated lipocalin on the 1st day after hospital admission, ng/mL	1.42 (1.05–2.28)	1.23 (0.2–1.76)	0.06
Serum neutrophil gelatinase-associated lipocalin on the 12th-14th day after hospital admission, ng/mL	1.93 (1.55–2.52)	1.53 (1.18–2.62)	0.031

**Fig. 1:**
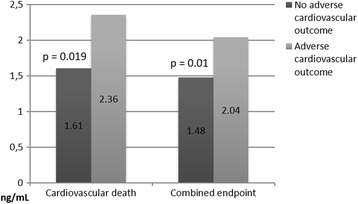
Medians of sNGAL levels on the 12th-14th day after hospital admission depending on cardiovascular outcome after 3 years of follow-up

**Fig. 2:**
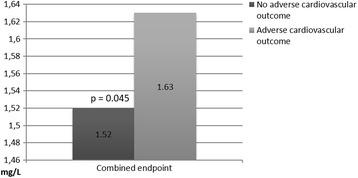
Medians of sCC levels on the 12th-14th day after hospital admission depending on cardiovascular outcome after 3 years of follow-up; the Y axis is cut from 0 to 1.46 for the better visualization of the results

An increased level of sNGAL on the 1st day after admission was significantly associated with in-hospital non-lethal cardiovascular complications [1.42 (1.17–2.27) and 1.20 (0.20–1.86) ng/mL in patients with and without them, respectively, *p* = 0.019]. Moreover, a higher level of sNGAL on the 12th-14th day after hospital admission was significantly associated with a cardiovascular death after 3 years of follow-up (Fig. 1). In addition, elevated concentrations of sCC and sNGAL on the 12th-14th day after hospital admission were significantly associated with a combined endpoint (Figs. 1 and 2). Notably, 132 patients had a level of sNGAL ≥ 1.25 ng/mL at hospital discharge that could possibly point on an infection [9]; however, none of them had signs or symptoms of any infectious disease both at hospital admission and hospital discharge. Nevertheless, we did not perform a specialized screening for latent infections.

For the determination of the independent factors of an adverse cardiovascular outcome, we performed a stepwise linear logistic regression. Factors included into regression were age, gender, past medical history of MI or stroke, diabetes mellitus, arterial hypertension, smoking, Killip class of acute heart failure at hospital admission, left ventricular ejection fraction (LVEF), localization of MI, number of affected coronary arteries, polyvascular disease, myocardial revascularization, sCC or sNGAL level on the 12th-14th day after hospital admission, and RD before hospital discharge. We identified anterior MI, LVEF < 40%, 3 affected coronary arteries, past medical history of stroke, level of sCC ≥ 1.9 mg/L on the 12th-14th day after hospital admission, level of sNGAL ≥ 1.25 ng/mL on the 12th-14th day after hospital admission, and RD before hospital discharge as the factors significantly associated with an adverse cardiovascular outcome after 3 years of follow-up (Table 6). Performance of PCI was associated with a significant decrease in risk of an adverse cardiovascular outcome (Table 6).

**Table 6:** Independent predictors of an adverse cardiovascular outcome after 3 years of follow-up

Predictor	*P* value	Odds ratio	95% confidence interval	
			Lower bound	Upper bound
Anterior localization of myocardial infarction	0.009	2.3	1.2	4.1
Left ventricular ejection fraction < 40%	0.001	3.6	1.7	7.6
Three affected coronary arteries	0.022	2.0	1.1	3.7
Past medical history of stroke	0.001	1.6	1.2	2.2
Level of serum cystatin C on the 12th-14th day after hospital admission ≥ 1.9 mg/L	0.004	1.9	1.2	2.9
Level of serum neutrophil gelatinase-associated lipocalin on the 12th-14th day after hospital admission ≥ 1.25 ng/mL	0.003	2.9	1.4	6.0
Renal dysfunction before hospital discharge	0.001	1.6	1.2	2.2
Percutaneous coronary intervention	0.001	0.4	0.3	0.7

**Fig. 3:**
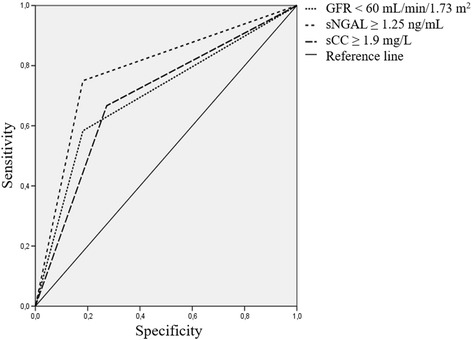
Comparison of the predictive value of the models based on different markers of renal dysfunction regarding adverse cardiovascular outcome

Finally, we compared AUC of the models based on RD before hospital discharge, level of sCC ≥ 1.9 mg/L, and level of sNGAL ≥ 1.25 ng/mL on the 12th-14th day after hospital admission. The latter model had the highest predictive value (AUC = 0.78) whilst two other models had equally lower predictive value (AUC = 0.70, Fig. 3).

## Discussion

In this study, we assessed the value of sCC and sNGAL level in prediction of an adverse cardiovascular outcome after STEMI. While sCC is a well-established marker of GFR [22, 23], sNGAL is mainly a neutrophil biomarker related to the bacterial infections; however, a number of studies also demonstrated an increase in sNGAL as a response to renal tubular damage [24–26]. Despite sNGAL is not well-recognized GFR marker compared to sCr and sCC and is not used for the calculation of GFR, sCr, sCC, and sNGAL all being the markers of renal tubular damage can be compared directly to each other for estimating efficiency in prediction of adverse outcome.

We previously demonstrated that sCC measured 1 day before and 7 days after CABG surgery is an appropriate predictor of in-hospital adverse cardiovascular and renal outcomes [27]. Here we showed that sCC and sNGAL can be potential markers of RD in patients with STEMI if measured on the 12th-14th day after hospital admission. Moreover, the level of sCC ≥ 1.9 mg/L and level of sNGAL ≥ 1.25 ng/mL on the 12th-14th day after hospital admission were associated with an adverse cardiovascular outcome in these patients after 3 years of follow-up. Out of three predictive models based on GFR < 60 mL/min/1.73 m^2^, level of sCC ≥ 1.9 mg/L, and level of sNGAL ≥ 1.25 ng/mL on the 12th-14th day after hospital admission, the latter had the highest predictive value.

This corresponds to the results of Akerblom et al. who identified high level of sCC as an independent predictor of cardiovascular death or MI in patients with acute coronary syndrome (ACS) after 1 year of follow-up [28]. In addition, our results are in accordance with the data of Lindberg et al. who detected that high level of sNGAL is an independent predictor of an adverse cardiovascular outcome after 2 years of follow-up in patients with STEMI who underwent PCI [29]. Noteworthy, one of the recent studies demonstrated that a multimarker approach using sCC and a number of other biomarkers added prognostic information to the GRACE risk score in patients with ACS and high risk defined by GRACE, with increasing 6-month mortality in patients with a higher number of elevated biomarkers at hospital admission [30].

In our study, 39 patients were lost to follow-up; however, all of them were alive at that moment. Out of them, 17 (43.6%) patients had major cardiovascular risk factors, i.e., diabetes mellitus, CKD, or arterial hypertension; 15 (38.5%) and 24 (61.5%) respectively had cardiovascular complications and a decrease in GFR during the hospital stay. All these variables were comparable to the general sample; hence, exclusion of the patients lost to follow-up from the statistical analysis was unlikely to affect the results. However, this still can be considered as a study limitation along with a single-center design.

Therefore, both sCC and sNGAL have high predictive value for the stratification of cardiovascular risk in patients with STEMI; however, sNGAL has an advantage over sCC.

## Conclusion

Patients with STEMI and in-hospital RD have higher levels of sCC and sNGAL compared to those without in-hospital RD. Elevated concentrations of sCC and sNGAL on the 12th-14th day after hospital admission can be suggested as the significant predictors of an adverse cardiovascular outcome in these patients after 3 years of follow-up. The model based on increased level of sNGAL has higher predictive value compared to those based on elevated concentration of sCC and decreased GFR.

## Abbreviations

ACS: Acute coronary syndrome
AUC: Area under the ROC curve
CABG: Coronary artery bypass graft
CAD: Coronary artery disease
CKD: Chronic kidney disease
ECA: Extracranial arteries
ESC: European Society of Cardiology
FDR: False discovery rate
GFR: Glomerular filtration rate
IMT: Intima-media thickness
LEA: Lower extremity arteries
LVEF: Left ventricular ejection fraction
MDRD: Modification of diet in renal disease
PCI: Percutaneous coronary intervention
RD: Renal dysfunction
ROC: Receiver operating characteristic
sCC: serum cystatin C
sNGAL: serum neutrophil gelatinase-associated lipocalin
STEMI: ST-segment elevation myocardial infarction
TLT: Thrombolytic therapy
